# Effect of Ultrasonic Intensity Treatment on the Physicochemical and Functional Properties of *Coregonus peled* Protamine

**DOI:** 10.3390/foods14030481

**Published:** 2025-02-02

**Authors:** Feifei Wang, Dong Shu, Yabo Wei, Xin Guo, Pingping Liu, Xiaorong Deng, Yunfeng Zhao, Yongdong Lei, Jian Zhang

**Affiliations:** 1 School of Food Science and Technology, Shihezi University, Shihezi 832003, China; 18892991858@163.com (F.W.); 20212111016@stu.shzu.edu.cn (D.S.); 18935813163@163.com (Y.W.); guoxin24yjs@163.com (X.G.); liupp0222@163.com (P.L.); dxr20099@163.com (X.D.); yunfeng_zhao@shzu.edu.cn (Y.Z.); 2 Key Laboratory of Agricultural Product Processing and Quality Control of Specialty (Co-Construction by Ministry and Province), School of Food Science and Technology, Shihezi University, Shihezi 832003, China; 3 Key Laboratory for Food Nutrition and Safety Control of Xinjiang Production and Construction Corps, School of Food Science and Technology, Shihezi University, Shihezi 832003, China; 4 Food Quality and Testing Center of Ministry of Agriculture and Rural Affairs, Xinjiang Academy of Agricultural and Reclamation Science, Shihezi 832000, China

**Keywords:** ultrasonic-assisted extraction, ultrasonic intensity, protamine, functional property

## Abstract

As the most valuable protein in the sperm of testes tissues of *Coregonus peled*, *Coregonus peled* protamine (CPP) had a natural antibacterial and antiseptic effect, but its physicochemical properties and functions are easily affected by the ultrasound-assisted extraction process. In this study, ultrasound-assisted extraction of CPP was used to investigate the effects of different ultrasonic intensities (0, 3.03, 6.07, 9.10, 12.13, and 15.16 W/cm^2^) on the structural and functional properties of CPP. The results showed that at moderate ultrasonic intensity (9.10 W/cm^2^), the protein was the most successful, as it was subjected to cavitation shear and microjet by ultrasound, which resulted in changes in protein structure, moderate unfolding of peptide chains, and changes in the secondary and tertiary structures of CPP. SEM images confirmed the changes in the microstructure of CPP. Ultrasound oxidized the proteins to varying degrees with the highest sulfhydryl and carbonyl and surface hydrophobicity content at an ultrasonic intensity of 9.10 W/cm^2^. At the same time, the solubility, antimicrobial activity, and heparin binding of CPP were affected. It is worth mentioning that the ultrasonicated CPP exhibited a stronger heparin-binding capacity compared to the non-ultrasonicated CPP. In conclusion, 9.10 W/cm^2^ was determined as the optimal ultrasonic intensity parameter for this study. In conclusion, the incorporation of appropriate ultrasonic intensity in the acidic extraction process could help to improve the functional properties of CPP, and ultrasound-assisted protein extraction has emerged as a reliable technique capable of modifying the structure and function of CPP.

## 1. Introduction

Protamine, the most medicinal and economically valuable protein from the seminal nest tissue, one of the fish by-products, is receiving more and more attention from researchers. Protamine is a naturally occurring cationic peptide rich in arginine and it can be classified as a monomer protamine, dimer protamine, and trimer protamine according to the different numbers of alkaline amino acids [1]. Protamine is soluble in water and dilute acid solution, and it has high temperature resistance, is bacteriostatic, and is non-toxic [2]. Therefore, it has been used in the food processing field for preservation of foodstuffs [3]. Currently, there are a number of methods for extracting fish protein, such as precipitation [4,5], ion exchange chromatography [1], and micellar extraction [6]. These methods have suffered from the real problems of low extraction rates, being time-consuming, and having high reagent consumption. In addition, relevant research on *Coregonus peled* protamine can provide an economically feasible solution to the environmental pollution and waste of resources caused by fish by-products.

Ultrasound, as a safe, efficient, and environmentally friendly technology, has been widely used to extract proteins from a variety of sources [7]. The advantage of ultrasound-assisted extraction technology is that ultrasound can promote cell wall rupture through the microjet formed by acoustic cavitation, thus significantly improving the efficiency of ultrasound extraction [8]. Meanwhile, ultrasound-assisted extraction can influence the molecular structure of target proteins by breaking hydrogen bonds or disrupting electrostatic interactions through cavitation effects [9]. However, in ultrasound-assisted technology, ultrasound temperature, ultrasound time, and ultrasonic intensity are all key process indicators that affect the ultrasound-assisted extraction of proteins. In the practical application of ultrasound technology, ultrasonic intensity, as a key factor affecting the extracted proteins, has to be taken into account. The synergistic effect between the key factors of ultrasound technology also affect the quality of the extracted target proteins and the processing technology solutions [10]. However, the synergistic effects among the factors affecting protein extraction by ultrasound have been less well studied and not clearly analyzed, and most of them are attributed to the combined effects of multiple factors, such as cavitation and shear produced by ultrasound. Therefore, researchers need to develop a more detailed discussion based on a careful exploration of the individual roles of each factor. In this study, based on previous studies related to ultrasound temperature on ultrasound-assisted extraction of CPP proteins, the same research method was used to continue to study the related effects of ultrasound intensity on CPP, which will lay a good foundation for subsequent in-depth discussion of the synergistic effects between ultrasonic intensity, ultrasound time, ultrasound temperature, and other factors. In addition, current research on ultrasonic intensity has focused on bovine liver peptides [11], skin collagen from albacore [12], and myofibrillar proteins from black soldier fly [13], etc., while there are fewer studies related to the effect of ultrasonic intensity on protamine. Therefore, in the present study, after investigating the effect of another important influencing factor (ultrasound time) on *Coregonus peled* protamine [14], we further explore the effect of ultrasonic power on the structural and functional properties of *Coregonus peled* protamine.

However, there is a key issue in ultrasonic intensity, which is how to reasonably control and select the appropriate ultrasonic intensity. This is mainly due to the fact that excessive ultrasound can have some drawbacks such as severe oxidation of proteins or lipids. This is caused by the formation of reactive free radicals as a result of localized high-energy, high-temperature cavitation bubbles [15]. Therefore, the objective of this study was to investigate the effects of ultrasonic intensity (0, 3.03, 6.07, 9.10, 12.13, and 15.16 W/cm^2^) on the physicochemical (e.g., secondary, tertiary, UV, and surface structure) and functional (e.g., bacteriostatic activity and heparin-binding capacity) properties of CPP from *Coregonus peled*. These results establish a foundation for the establishment of a new method for the extraction of CPP, which provides a pathway to promote the development and utilization of protamine in the food sector.

## 2. Materials and Methods

### 2.1. Coregonus Peled Nesting Material

*Coregonus peled* sperm nest tissues were supplied by Sail Lake Fisheries Technology Development Co. (Bortala Mongol Autonomous Prefecture, Xinjiang, China). Detailed pre-processing of spermathecal tissues in *Coregonus peled* was consistent with our previous study [14], such as the process of cleaning, removing impurities, selecting, rinsing, and weighing. The processed raw material was encapsulated into 100 g/bag with sealing bag and stored at −20 °C for spare use.

### 2.2. Extraction

The extraction method of CPP followed the method of Liu et al. [6], and it was similar to the extraction method in our previous study [14]. Briefly, sperm nest tissues were extracted by ultrasound-assisted acid extraction; SDS (sodium dodecyl sulfate) was added and adjusted to a concentration of 40 mM, magnetically stirred for 30 min, and left to stand and centrifuged; and the precipitate produced was retained. The precipitate continued to be dissolved using propanol. It was then titrated using 0.14 M NaCl until white precipitates appeared and then it continued to titrate, was centrifuged after no precipitate appeared, and the precipitate was collected and freeze-dried to obtain CPP powder for spare use. The specific process parameters were as follows: the concentration of sulfuric acid was 0.15 M, the liquid-to-feed ratio was 3:1, the ultrasonic frequency was 20 kHz (ultrasonic cell breaker, JY98-IIIDN, Ningbo Science Biotechnology Co., Ltd., Ningbo, China), and the ultrasonic intensities of 0, 3.03, 6.07, 9.10, 12.13, and 15.16 W/cm^2^ were used; the extraction was repeated 3 times at 30 °C for 30 min. The calculation method of ultrasonic intensity was calculated with reference to Suchintita [10], as shown below (Equation (1)):

(1) UI=P/S

The equation is defined as follows: UI represents the ultrasonic intensity (W/cm^2^), P is the ultrasonic power (W), and S denotes the area of the transducer’s emitting surface (cm^2^).

### 2.3. Protein Yield

The CPP protein yields obtained at different ultrasonic intensities were calculated as below (Equation (2)):

(2) $$Protein\;yield\;\left(\%\right)=W_{P}\times C_{P}/W_{T}\times C_{T}\times 100$$

Here, W_P_ denotes the weight of CPP (g), C_P_ represents the concentration of CPP (g/L), W_T_ refers to the dry weight of the spermatheca tissue of *Coregonus peled* (g), and C_T_ indicates the protein concentration of the spermatheca tissue of *Coregonus peled* (g/L).

### 2.4. Secondary Structure

CPP samples treated with different ultrasonic intensities were processed and measured using the method of Wang [14]. Briefly, the sample was KBr = 1:100 and the spectral range was 4000–400 cm^−1^ (Fourier Infrared Spectrometer, FTIR, Thermo Fisher, Thermo Fisher Scientific, Waltham, MA, USA). The infrared spectra of each CPP sample in the protein amide I band, typically observed in the range of 1700–1600 cm^−1^, were resolved, and the relative amounts of secondary structure were expressed as the relative percentages of *α*-helix, *β*-sheet, *β*-turn, and random curl.

### 2.5. Tertiary Structure

The method used was that of Jiang [16], with modifications, and the specific parameters in this study were consistent with those of previous studies [14]. The excitation wavelength was 295 nm and the slit width and sensitivity were both 5.5 mm.

### 2.6. Ultraviolet (UV) Absorption Spectra

UV spectral analysis was performed as reported by Mach et al. [17]. The UV absorption spectra of all samples were scanned and then analyzed at room temperature using a UV–visible spectrophotometer (model: T3200, Shanghai Youke Instrument Co., Ltd., Shanghai, China).

### 2.7. Scanning Electron Microscope (SEM)

The SEM analysis of CPP was conducted following a modified version of the method described by Wei [18], with minor adjustments. CPP samples were scanned and imaged at an acceleration voltage of 50 kV and a magnification of 400×.

### 2.8. The Functional Properties of CPP

#### 2.8.1. Solubility

Solubility was determined using the method described by Zhang et al. [19] with slight variations and consistent with the conditions of previous studies [14]. The protein volume concentration was adjusted to 100 mg/mL. The assay was carried out using Kohler’s Brilliant Blue kit (Beyotime Biotech Inc., Shanghai, China) and PBS was used as the blank control. Protein solubility was calculated as shown below (Equation (3)):

(3) $$Solubility\;(\%)=P_{S}/P_{W}\times 100$$

where P_S_ represents the protein concentration of the supernatant (mg/mL) and P_W_ denotes the protein concentration of the whole solution (mg/mL).

#### 2.8.2. Carbonyl, Sulfhydryl, and Surface Hydrophobicity

Carbonyl, sulfhydryl, and surface hydrophobicity contents were determined by the same methods as in previous studies [14]. The method for determining the carbonyl content was briefly described as below. A total of 3 mL CPP solution (4 mg/mL) and 2,4-Dinitrophenylhydrazine (DNPH, 10 mM) were obtained, the reaction time was 1 h at 25 °C, and 3 mL trichloroacetic acid (TCA, 20%) was added. After centrifugation (5000 r/min, 10 min, 4 °C), the precipitation was retained and washed with 1 mL ethanol/ethyl acetate (1:1, *v*/*v*) 3 times. The residue was dissolved with 3 mL trichloroacetic acid and then centrifuged after bathing at 37 °C for 15 min. The content of carbonyl group was determined with the supernatant at 370 nm. The sulfhydryl content was determined as below. A total of 0.3 mL of β-mercaptoethanol was added in 1.8 mL of 5 mg/mL CPP solution and incubated for 1 h at 25 °C. The incubation was continued with 10 mL of TCA solution (12%) for 1 h. Centrifugation (25 °C, 3000 r/min, 10 min) was performed. The precipitate was washed twice with TCA solution and the residue was dissolved with 1 mL SDS-Trisbuffer (5% SDS, 0.1 M Tris, pH 8.0). The solution (0.75 mL) was mixed with SDS-Trisbuffer (3.0 mL) and 5,5′-Dithiobis-(2-nitrobenzoic acid) (DTNB, 0.3 mL) was added. The reaction was carried out in the absence of light for 30 min and the absorbance was 412 nm. The determination of surface hydrophobicity was described as below. We dissolved CPP with PBS (10 mM, pH 7.0) and adjusted the concentration (0.2–1.0 mg/mL). Each concentration of CPP solution (8 mL) was mixed with 8-aniline-1-naphthalene sulfonic acid (ANS, 100 μL, 8 mM) and the reaction was determined after 2 min of light avoidance.

#### 2.8.3. Zone of Inhibition

The zone of inhibition was in line with the method of Tao with slightly different conditions [20]. Experiments were carried out according to the method of the previous study [14]. The concentration of the bacterial solution was 10^6^–10^8^ CFU/mL, the concentration of the sample protein was 30 mg/mL, the volume of the single-well protein addition was 200 μL, the zone of inhibition was observed and measured after 24 h of incubation at 37 °C, and the diameters were averaged three times. Each set of experiments was performed 3 times in parallel. The commercially available protamine (Protamine, Merck, Germany) was used as a control and the zone of inhibition was calculated as 1. The zone of inhibition of the rest of the samples was calculated by the same visualization. *E. coli* (CICC 21530), *Salmonella Typhimurium* (ATCC 14028), and *S. aureus* (ATCC 25923) were provided by the Analysis and Testing Center of Xinjiang Academy of Agricultural and Reclamation Sciences.

#### 2.8.4. Heparin Binding

The parameters were determined with reference to the method of Jaques [21] and a previous study [14]. The absorbance was measured at a wavelength of 500 nm. Heparin sodium (85 U/mL) solution was added dropwise to a quartz cuvette containing 2.5 mL of the homogeneous sample until a sharp increase in absorbance was observed. The volume of drops added was accurately recorded and used for calculation. The formula was as below (Equation (4)):

(4) $$Measured\;potency\;(IU/mg)=V_{T}\times C_{T}/V_{S}\times C_{S}$$

where V_T_ denotes the volume of sodium heparin consumed (mL), V_S_ represents the volume of the sample to be tested (mL), C_T_ is the concentration of sodium heparin (IU/mL), and C_S_ denotes the concentration of the solution to be tested (mg/mL).

### 2.9. Statistical Analysis

Samples were assayed in three parallels and kept for ANOVA and Duncan’s test (*p* ≤ 0.05). Data were processed using IBM SPSS Statistics software (version 22.0) and plotted using Origin2024 software (version 2024b). Spectral data were analyzed using OMNIC (version 9.2.7) and Peakfit software (version 4.12). SEM was combined using Photoshop software (version 2021).

## 3. Results and Discussion

### 3.1. Physical and Chemical Properties

#### 3.1.1. Protein Yield

**Figure 1:**
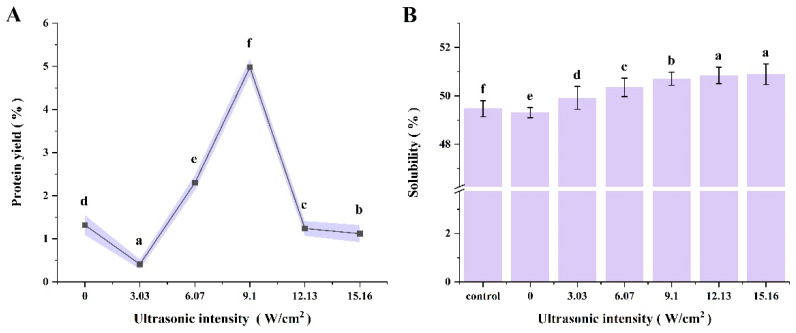
Changes in ultrasonic intensity and their impact on (**A**) yield and (**B**) solubility. Different letters (a–f) indicate significant differences (*p* ≤ 0.05).

As shown in Figure 1A, protein yield increased with increasing ultrasonic intensity, reaching a maximum of 4.98 ± 0.20% at 9.10 W/cm^2^ (*p* ≤ 0.05). This suggested that an optimal ultrasonic intensity could enhance the protein yield. Appropriate ultrasonic intensity can increase the protein yield. The main reason is that the physical, mechanical, and chemical effects of acoustic cavitation can increase the protein release rate [22]. However, as the ultrasonic intensity increased to 12.13 W/cm^2^, the protein yield decreased significantly. This decrease may be due to the fact that excessive ultrasound-generated energy destroyed the protein active sites and protein aggregation produced non-covalent interactions [23]. At the same time, the cavitation effect in the extraction system was enhanced and the mass transfer effect was weakened, which reduced the protein yield [24]. When the ultrasonic intensity was further increased to 15.16 W/cm^2^, the protein yield stabilized, which was similar to the trend observed by Wu and Ni [13,25]. In their studies, increasing the power initially increased the total flavonoids and myofibrillar protein extraction rate, but the extraction rate stabilized beyond 700 W and 300 W. Our results were consistent with these findings, indicating that 9.10 W/cm^2^ is the optimal ultrasonic intensity for extracting CPP.

#### 3.1.2. Secondary Structure

**Figure 2:**
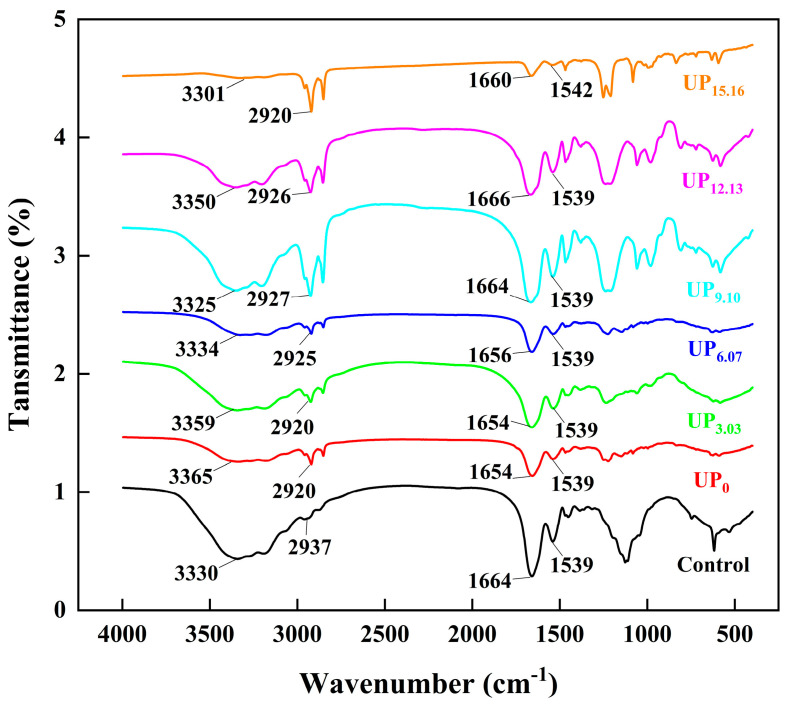
Changes in ultrasonic intensity and their impact on infrared spectra of CPP proteins during ultrasound-assisted acidic extraction. The control was a commercially available standard and UP indicates samples treated at each ultrasonic intensity.

As shown in Figure 2, all the samples showed distinct absorption peaks which were observed in the range of 3600–3200 cm^−1^ (amide A band) and were mainly characteristic peaks caused by the stretching vibrations of intermolecular hydrogen between N-H and O-H groups [26]. The absorption peaks in the ultrasound group (3.03, 6.07, 9.10, 12.13, and 15.16 W/cm^2^) changed from 3359 cm^−1^ to 3301 cm^−1^ compared to the standard control group, which may be due to inter- and intramolecular interactions to some extent [27]. The absorption peaks in the amide I band (1700–1600 cm^−1^) were attributed to the stretching vibrations of the C=O bond and C-N bonds [28]. Meanwhile, the absorption peaks in the amide II band (1600–1500 cm^−1^) and amide III band (1330–1220 cm^−1^) were primarily attributed to C-N stretching vibration and N-H bending. The intensity of the absorption peaks in the amide II band gradually increased as the ultrasonic intensity was increased to 9.10 W/cm^2^, compared to the standard control. When the ultrasonic intensity was further increased to 12.10 W/cm^2^ and 15.16 W/cm^2^, the intensity of the absorption peaks gradually decreased, indicating that ultrasonic treatment changing the secondary structure of the CPP protein was modified to some extent.

**Figure 3:**
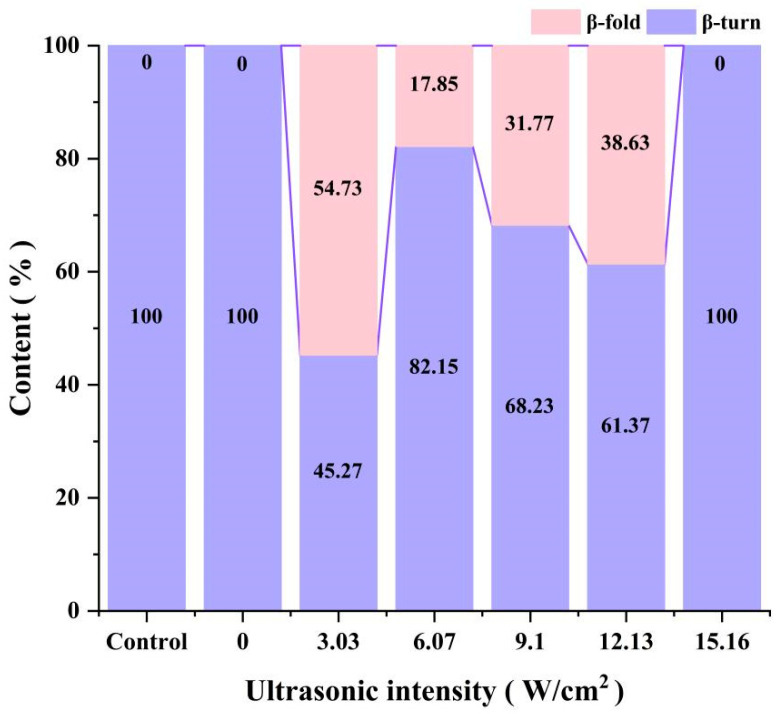
The effect of ultrasonic intensity on the secondary structure of CPP during UA acid extraction.

It is worth noting that the amide I band (1600–1700 cm^−1^) was very sensitive to changes in secondary structure and Figure 3 provides the percentage of *α*-helix, *β*-sheet, *β*-turn, and the random coil secondary structures in CPP. All samples were free of *α*-helix and the random coil structures. This may be related to the high basic amino acid content of protamine [1,29]. Both the standard control and the CPP proteins produced using the method without the introduction of sonication only had *β*-turn structures. After the introduction of ultrasound-assisted extraction, however, with the increase in ultrasonic intensity (3.03, 6.07, 9.10, and 12.13 W/cm^2^), the *β*-sheet structure appeared in the secondary structure and the content of the *β*-sheet structure decreased and then increased. This may be due to the fact that the hydrogen bonds between the polypeptide chains of the proteins were broken by ultrasound and the peptide chains partially unfolded, which made some of the stable *β*-turn structures transform into unstable *β*-sheet structures. It was similarly demonstrated that ultrasonic treatment of proteins resulted in a decrease in *α*-helix content, an increase in *β*-sheet content, and an increase in protein flexibility by Wang [30] and Ni [13]. When the ultrasonic intensity was increased to 15.16 W/cm^2^, the *β*-sheet structure disappeared and the secondary structure composition showed the same results as in the control and non-ultrasound-treated groups. This may be due to the fact that high-intensity sonication severely damaged the structure of the protein and the peptide chains were partially broken and then aggregated and reassembled to form new aggregates, resulting in the loss of the unique functional activity of the CPP protein. This was corroborated with the results of SEM and heparin titration below.

#### 3.1.3. Tertiary Structure

**Figure 4:**
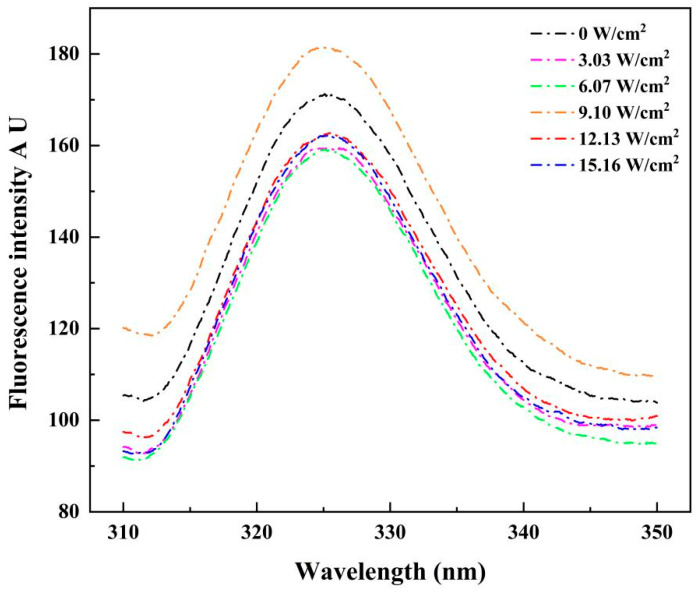
Changes in ultrasonic intensity and their impact on endogenous fluorescence spectra of CPP proteins during ultrasound-assisted acidic extraction. UP indicates samples treated at each ultrasonic intensity.

Endogenous fluorescence is a commonly used technique for testing reactions to the tertiary structure of proteins, relying mainly on Trp (tryptophan)/Tyr (tyrosine). The intensity of the fluorescence emitted was sensitive to polarity or the local environment [9]. The tertiary structure of CPP extracted at different ultrasonic intensities was evaluated using endogenous fluorescence, as shown in Figure 4. The intrinsic fluorescence spectra were similar across all samples, with the maximum absorption peak observed at 325 nm. The fluorescence intensity was maximum when the ultrasonic intensity was 9.10 W/cm^2^, which was higher than that of the group without the use of ultrasound treatment. This was due to the fact that as the ultrasound energy increased, the energy generated by ultrasound caused the protein structure to unfold moderately and the tryptophan residues on the surface of the protein were exposed. However, the fluorescence intensity gradually decreased when the ultrasonic intensity was too high. This may be due to the oxidation of the Trp fraction [31], which may also be the result of excessive ultrasound energy causing the protein structure to fold, disrupting protein hydrophobic interactions and reducing the exposure of amino acid residues on the outside of the protein [32]. In addition, excessive ultrasound energy may lead to the folding of the protein structure, the formation of polymers, and a reduction in fluorescence intensity [33], which would be similar to previous findings [14]. Similar reductions were found for endogenous fluorescence of myofibrillar proteins from black soldier fly and black bean proteins by Ni [13] and Jiang [34].

#### 3.1.4. UV Full-Wavelength Scanning

**Figure 5:**
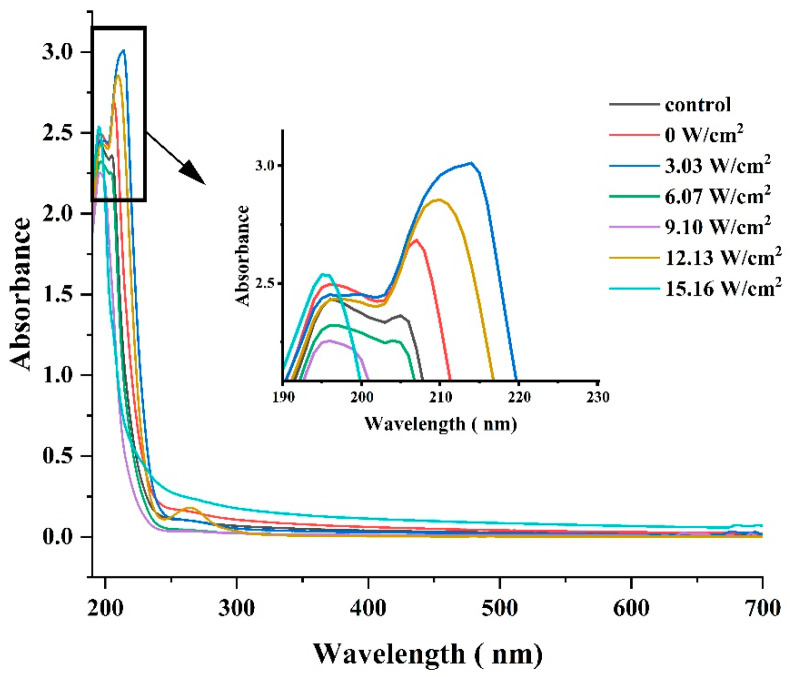
Changes in ultrasonic intensity and their impact on UV full-wavelength scanning of CPP proteins during ultrasound-assisted acidic extraction. Control was a commercially available standard.

The variation in the UV spectra of CPP extracted using different ultrasonic intensities is shown in Figure 5. The highest absorption peak near 210 nm was associated with the carbonyl, carboxyl, and amide groups, associated with triple helices in the polypeptide chain of protamine [35]. All of the samples of CPP showed absorption peaks around 196 nm, which is consistent with the results observed in Thunnus alalunga collagen [13]. At ultrasonic intensities of 12.13 and 15.16 W/cm^2^, there was only an absorption peak at 196 nm, which could be attributed to the fact that ultrasonic waves generated by excessive ultrasonic intensities caused structural changes in the protein structure of CPP, which resulted in refolding and irreversible damage, exhibiting aggregation and gelling, and yielding similar results. The structural changes in the proteins caused changes in the relevant functional properties, which was also confirmed by the results of the bacteriostatic and heparin titration experiments below. In addition, a weak absorption peak was observed at 260 nm, suggesting that the tryptophan and tyrosine content in CPP was relatively low. This finding was in general agreement with that of Ni et al. [13].

#### 3.1.5. SEM Surface Morphology Analysis

**Figure 6:**
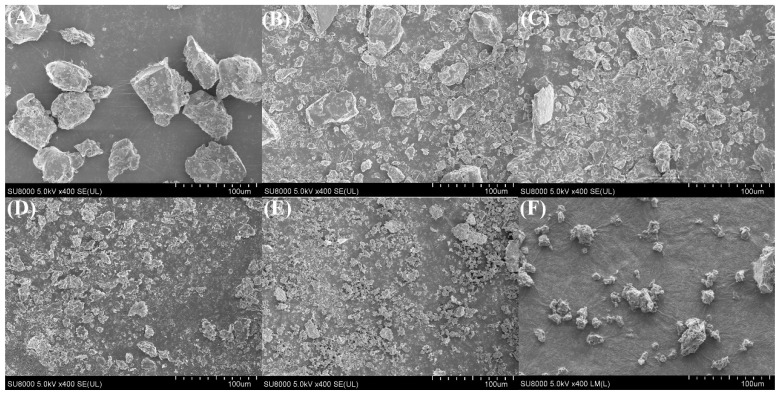
Effect of ultrasonic intensity on the change in SEM apparent morphology of CPP protein during ultrasound-assisted acidic extraction, where A–E represent samples with ultrasonic intensities: (**A**) 0 W/cm^2^, (**B**) 3.03 W/cm^2^, (**C**) 6.07 W/cm^2^, (**D**) 9.10 W/cm^2^, and (**E**) 12.13 W/cm^2^; (**F**) 15.16 W/cm^2^.

Figure 5 showed the SEM images of CPP at different ultrasonic intensities. As shown in Figure 6A, the CPP powder without ultrasound-assisted extraction (0 W/cm^2^) had a clear blocky structure with well-defined edges. With the introduction of ultrasound-assisted technology (Figure 6B–F), the CPP powder showed an overall fragmentation trend. When the ultrasonic intensity was low (Figure 6B,C), the CPP powder was gradually fragmented, with a decrease in the blocky structure and an increase in the tiny particles. When the ultrasonic intensity was increased to 9.10 W/cm^2^ (Figure 6D), the CPP fragmented structure was more uniform. When the ultrasonic intensity was further increased to 12.13 W/cm^2^, the degree of CPP powdering increased further, and small irregular particles with some adherent aggregates appeared. When the ultrasonic intensity was increased to 15.16 W/cm^2^, the CPP showed different sizes, irregular shapes, and uneven surfaces. The particles of the samples in the ultrasonication group (Figure 6B–F) were smaller than those in the control group (Figure 6A), suggesting that the localized microjet and cavitation formed by ultrasonication reduced the particle size of the protein, which was similar to the results of the study on whey protein [36]. However, at an ultrasonic intensity of 15.16 W/cm^2^, the larger ultrasound energy severely disrupted the secondary structure and tertiary structure of the protein, resulting in samples exhibiting irregular aggregation and adhesion. This was corroborated with the results of the bacteriostatic and heparin-binding assays later on. The study of Hu on ultrasound of collagen had similar results, i.e., ultrasonication significantly disrupted the natural distribution and arrangement of collagen proteins [37]. The results of Pan et al. also showed that ultrasonic power produced significant physical changes in the microstructure of collagen [12].

### 3.2. Functional Nature of the CPP

#### 3.2.1. Solubility

The solubility of CPP at different ultrasonic power intensities is shown in Figure 1B. With the increase in ultrasonic intensity, the solubility first increased and then remained stable, and the CPP protein solubility was maximal when the ultrasonic intensity was 9.10 W/cm^2^. The cavitation and local microjet generated by ultrasound caused the protein structure to be damaged and unfolded to different degrees, which led to the dissociation of CPP into smaller soluble aggregates and increased the solubility of the protein [14,38]. This result was corroborated by the analysis of the 3.1.5 SEM results above, where the smaller the particle size, the higher the solubility.

#### 3.2.2. Carbonyl, Sulfhydryl, and Surface Hydrophobicity

**Figure 7:**
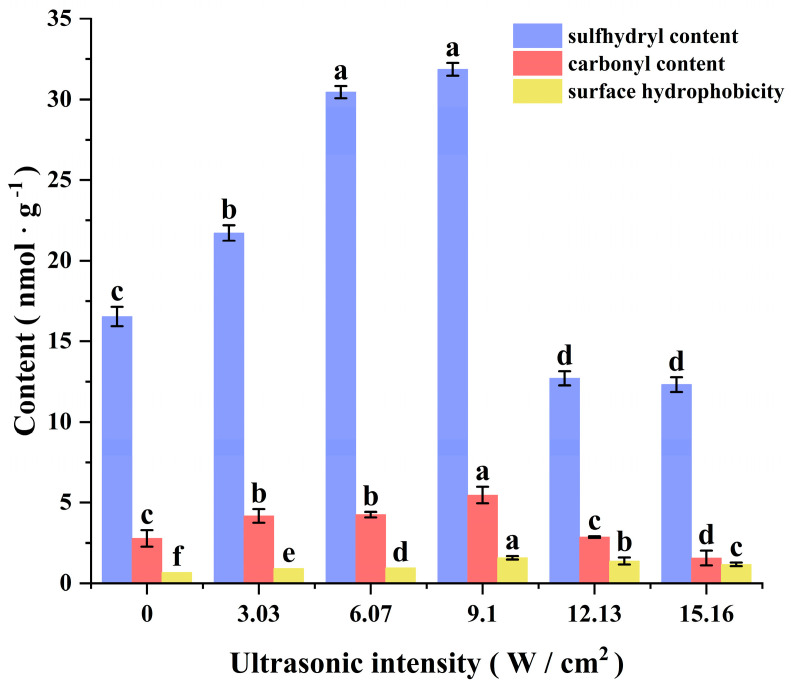
Effects of different ultrasonic intensity treatments on sulfhydryl, carbonyl, and surface hydrophobicity of CPP. Letters (a–f) indicate significant differences (*p* ≤ 0.05).

Sulfhydryl, carbonyl, and surface hydrophobicity were important indicators of the degree of protein oxidation during sonication. As shown in Figure 7, the sulfhydryl content increased and then decreased with increasing ultrasonic intensity. The sulfhydryl content was highest when the ultrasonic intensity was 9.10 W/cm^2^. This may be due to the fact that when the ultrasonic intensity was low, the protein peptide chain was moderately unfolded under the influence of acoustic vibration and cavitation, and more sulfhydryl buried inside the protein was exposed. With the increase in ultrasonic intensity, the sulfhydryl content was significantly reduced and stabilized, which may be due to the fact that the excessively high ultrasonic intensity severely damaged the structure of the protein peptide chain, which led to aggregation of proteins, and some of the sulfhydryl groups were re-embedded into the proteins; at the same time, the excessively high ultrasonic intensity accelerated the sulfhydryl oxidation and re-embedding process [13]. The variation in carbonyl content remained basically the same as that of sulfhydryl. The highest carbonyl content was observed at an ultrasonic power intensity of 9.10 W/cm^2^. This may be due to protein oxidation triggered by highly reactive free radicals generated by cavitation. When the ultrasonic intensity was further increased, the carbonyl content decreased, which was in agreement with the results of Ni, indicating that protein oxidation was delayed [13]. It is possible that excessive sonication caused severe disruption of the protein structure and excessive exposure of tryptophan residues on the surface of the protein, thereby enhancing the surface hydrophobicity and antioxidant properties of the protein and thus producing such interesting results [39]. And the trend of surface hydrophobicity also confirmed this view. As shown in Figure 7, the surface hydrophobicity of ultrasonicated CPP was generally higher than that of the un-ultrasonicated CPP and reached the maximum at 9.10 W/cm^2^. This indicates that the energy generated by the moderate ultrasonic intensity partially unfolded the protein structure, and the hydrophobic amino acids that originally folded inside the protein were more exposed [14]. With the increase in ultrasonic intensity, the over-intense ultrasound waves severely damaged the structure of the proteins, causing a certain degree of denaturation and aggregation of the proteins, resulting in some of the hydrophobic residues being re-buried into the proteins, and ultimately reducing the surface hydrophobicity of the proteins. Ni et al. showed similar results to those of the present study [13].

#### 3.2.3. Zone of Inhibition

**Figure 8:**
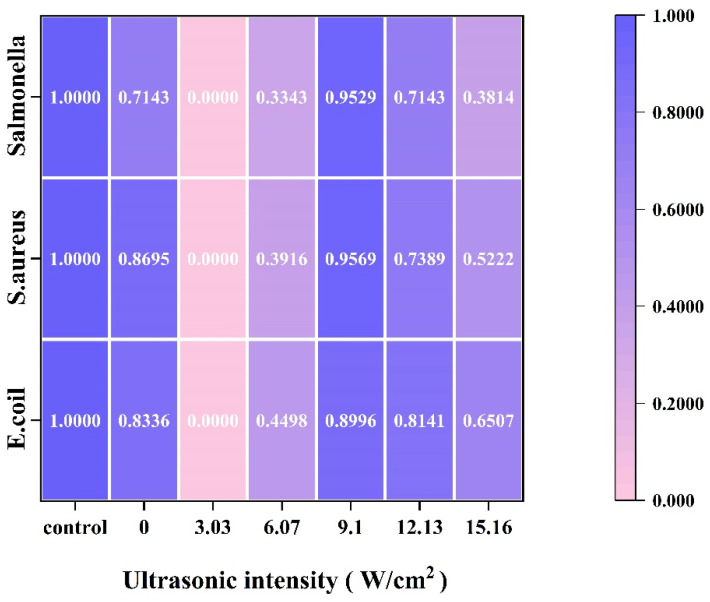
Effect of ultrasonic intensity on the bacteriostatic activity of CPP proteins during UA acid extraction. Control was a commercially available standard.

The size of the inhibition circle was a common factor in indicating the magnitude of the inhibitory activity of CPP proteins. As shown in Figure 8, using a heat map to represent the inhibition effect, we defined the diameter of the inhibition circle of the standard as 1, and the inhibition effect was obtained by normalizing the rest of the treatment groups (ultrasonic intensity: 0, 3.03, 6.07, 9.10, 12.13, and 15.16 W/cm^2^). The better the bacteriostatic effect, the darker the color. As shown in Figure 5, with the increase in ultrasonic intensity, the bacteriostatic activity of CPP first increased and then decreased. When the ultrasonic intensity was low (<9.10 W/cm^2^), the bacteriostatic effect of the CPP protein was poor and even weaker than the bacteriostatic property of CPP (0 W/cm^2^) in the group without ultrasonic treatment. This might be due to the fact that at lower ultrasonic intensities, less energy was generated, the solution in the reaction system was less perturbed by the energy, thus less cavitation and microjets were generated, and the structure of the CPP protein was less affected. When the ultrasonic intensity was 9.10 W/cm^2^, the inhibition of the three bacteria in each treatment group showed strong inhibition, but this was slightly lower than the standard control group. This might be due to the fact that the increase in ultrasonic energy led to a moderate unfolding of the structure of protamine and a greater leakage of its arginine residues, which enhanced the destruction of the bacterial cell wall [40]. The bacteriostatic activity of CPP proteins gradually decreased with a further increase in ultrasonic intensity. This might be due to excessive ultrasound energy, resulting in changes in the protein structure. The inhibitory activity of the 9.10 W/cm^2^ ultrasonic intensity group, which had the strongest inhibitory activity among the ultrasound treatment groups, was slightly lower than that of the standard control group. This might be due to the fact that the purity of the CPP was affected by the extraction method, which was lower than the purity of the purchased standard protamine. In addition, CPP showed stronger inhibitory activity against S. aureus and Salmonella than E. coli, which was in agreement with the findings of Lesmes et al., showing that the inhibitory effect of protamine on Gram-positive bacteria was more pronounced [41].

#### 3.2.4. Heparin Binding

**Figure 9:**
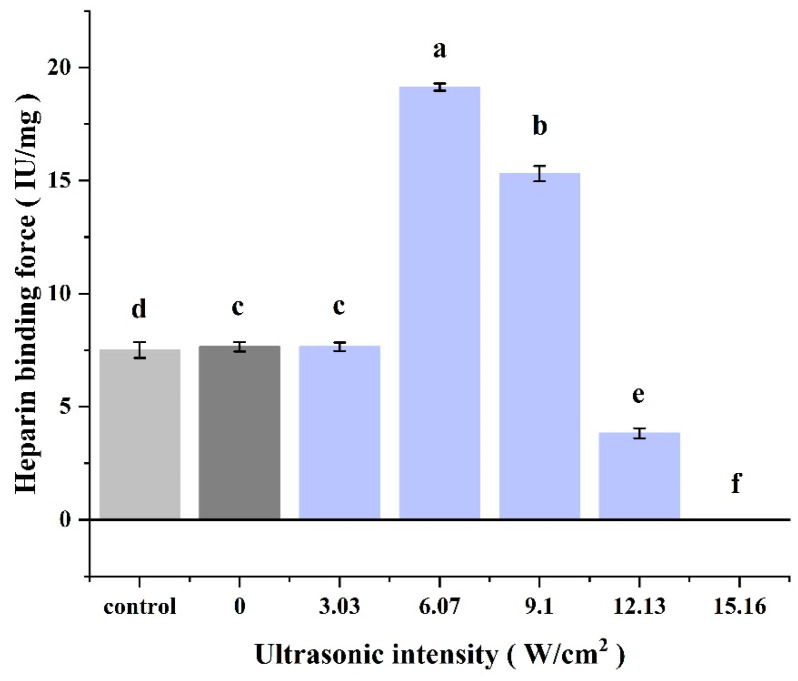
Effect of ultrasonic intensity on the heparin binding of CPP proteins during UA acid extraction. Control was a commercially available standard. Different letters (a–f) indicate significant differences (*p* ≤ 0.05).

Heparin sodium titration is a method used to evaluate the bio-efficacy of CPP proteins. As shown in Figure 9, the heparin-binding capacity of CPP increased initially and then decreased with increasing ultrasonic intensity. At ultrasonic intensities of 0 and 3.03 W/cm^2^, the heparin-binding capacity was similar to the results of the standard control. When the ultrasonic intensity was increased to 6.07 W/cm^2^, the heparin-binding capacity reached its maximum. When the ultrasonic intensity was further increased to 9.10 W/cm^2^, the heparin-binding force decreased, but was still significantly higher than that of the standard control group. When the ultrasonic intensity was further increased to 12.13 W/cm^2^, the heparin-binding capacity sharply reduced and was even lower than that of the standard control group and group 0 W/cm^2^. When the ultrasonic intensity was increased to 15.16 W/cm^2^, the CPP protein lost its heparin-binding capacity. This was similar to the trend of the results of the study on inhibitory effects above, and also similar to the results of the previous study [14].

## 4. Conclusions

This study systematically examined the effects of varying ultrasonic intensities on the structural and functional properties of CPP. The findings revealed that moderate ultrasonic intensity induced conformational changes and structural unfolding in the protein through cavitation effects and excitation. These changes altered the intrinsic fluorescence and Fourier Transform Infrared (FTIR) spectra, caused fragmentation of CPP powder particles, promoted protein oxidation, and enhanced the solubility, bacteriostatic activity, and heparin-binding capacity of CPP. In conclusion, these findings confirmed that appropriate ultrasonic intensity treatment (9.10 W/cm^2^) was effective in increasing protein yield and altering the structure and function of CPP. This study offered valuable insights into the specific effects and underlying mechanisms of ultrasonic treatment on the structural and physicochemical properties of CPP. In summary, ultrasound-assisted extraction of CPP has had breakthroughs and application prospects in many fields such as food processing, food preservation and freshness, and pharmaceutical product development. It has also established a scientific and theoretical foundation for the comprehensive application of CPP in modern production, offering a sustainable development pathway for the large-scale utilization of protamine. When applying ultrasound-assisted extraction of proteins, choosing the most suitable power intensity and avoiding the potential side effects of strong ultrasound are crucial for production. In addition, the mechanism and quantitative analysis of the effect of ultrasound on the microstructure of CPP still need more in-depth studies by researchers.

## Data Availability

The data presented in this study are available upon request from the corresponding author. The data are not publicly available due to privacy restrictions.
